# Fabrication and Highly Efficient Dye Removal Characterization of Beta-Cyclodextrin-Based Composite Polymer Fibers by Electrospinning

**DOI:** 10.3390/nano9010127

**Published:** 2019-01-20

**Authors:** Rong Guo, Ran Wang, Juanjuan Yin, Tifeng Jiao, Haiming Huang, Xinmei Zhao, Lexin Zhang, Qing Li, Jingxin Zhou, Qiuming Peng

**Affiliations:** 1 State Key Laboratory of Metastable Materials Science and Technology, Yanshan University, Qinhuangdao 066004, China; guorong@stumail.ysu.edu.cn (R.G.); pengqiuming@ysu.edu.cn (Q.P.); 2 School of Environment and Civil Engineering, Dongguan University of Technology, Dongguan 523808, China; huanghaiming52hu@163.com; 3 Hebei Key Laboratory of Applied Chemistry, School of Environmental and Chemical Engineering, Yanshan University, Qinhuangdao 066004, China; wr1422520780@163.com (R.W.); jjy1729@163.com (J.Y.); zhaoxinmei2008@yeah.net (X.Z.); zhanglexin@ysu.edu.cn (L.Z.); zhoujingxin@ysu.edu.cn (J.Z.)

**Keywords:** beta-cyclodextrin polymer, host–guest interaction, dye removal, wastewater treatment, electrospinning

## Abstract

Dye wastewater is one of the most important problems to be faced and solved in wastewater treatment. However, the treatment cannot be single and simple adsorption due to the complexity of dye species. In this work, we prepared novel composite fiber adsorbent materials consisting of ε-polycaprolactone (PCL) and beta-cyclodextrin-based polymer (PCD) by electrospinning. The morphological and spectral characterization demonstrated the successful preparation of a series of composite fibers with different mass ratios. The obtained fiber materials have demonstrated remarkable selective adsorption for MB and 4-aminoazobenzene solutions. The addition of a PCD component in composite fibers enhanced the mechanical strength of membranes and changed the adsorption uptake due to the cavity molecular structure via host–guest interaction. The dye removal efficiency could reach 24.1 mg/g towards 4-aminoazobenzene. Due to the admirable stability and selectivity adsorption process, the present prepared beta-cyclodextrin-based composite fibers have demonstrated potential large-scale applications in dye uptake and wastewater treatment.

## 1. Introduction

Contamination by dyes has led to many environmental problems [1,2,3,4,5,6,7,8,9]. In recent years, how to manage water pollution by an efficient, simple, and safe method has become a hot topic in the field of wastewater treatment research [10,11,12]. However, the treatment of dye wastewater is much more difficult than other kinds of wastewater due to the complexity and diversity of dye molecules [13,14,15,16,17,18,19,20,21]. Azo dyes are one of the most widely used dyes with chromogenic groups [22,23,24]. Azo dyes and their byproducts have been a focus of research attention due to their severe toxicological effects on human health: they are known to be genotoxic agents with carcinogenic properties [25,26,27,28] that may lead to birth defects [29] and food security issues [30,31,32,33]. Beyond this, dysfunction of the kidney, reproductive system, liver, brain, and central nervous system could also be exacerbated by azo dyes [34,35,36]. Thus, it is urgent that we learn how to properly deal with Azo dyes to achieve a safe and clean environment. Many studies have made great efforts to do this [37,38,39,40]. However, traditional technologies and methods cannot deal with the fact that different kinds of dyes need different treatments. Azo dyes are no exception. Therefore, selective chemical adsorption for different dyes has attracted wide interest. The selectivity of adsorbents is the key to the adsorption capacity and performance.

On the other hand, cyclodextrin, as a class of cyclic oligosaccharides, has a hollow circular cone with external hydrophilicity and internal hydrophobicity. This special structure has many special physical and chemical properties. It can selectively bind small organic molecules in an aqueous solution and the inclusion complexes formed have different degrees of stability [41,42,43]. Therefore, cyclodextrins and their derivatives are widely used in medicine, food, chemical engineering materials, and especially in wastewater treatment [44,45,46,47,48]. For example, Li et al. prepared a cyclodextrin-based material to remove malachite green [49]. The adsorption results fit the Langmuir model and the maximum adsorption capacity reached 91.9 mg/g. Yilmaz et al. synthesized two β-cyclodextrin-based polymers with the help of 4,4′-methylene-bis-phenyldiisocyanate (MDI) or hexamethylenediisocyanate (HMDI) [50]. These materials could remove azo dyes, as well as aromatic amines, while the dominant adsorption mechanism was host–guest interaction. Ozmen et al. synthesized three beta-cyclodextrins and a starch-based polymer using HMDI [51]. They have compared the adsorption capacity and the results showed high adsorption performance toward some azo dyes. Generally, researchers have made a lot of efforts towards dealing with azo dyes in the field of wastewater treatment. In addition, electrospinning technology demonstrated an effective method to prepare fiber materials due to its obvious advantages of simple operation and easy regulation [52,53,54,55,56]. At present, some cyclodextrin-based fiber systems via electrospun approach have been reported [57,58,59,60]. For examples, Cui et al. described the investigation of plasma-treated poly(ethylene oxide)-beta-cyclodextrin nanofibers to enhance the antibacterial activity [57]. Celebioglu et al. demonstrated the electrospinning of polymer-free nanofibrous structures from an inclusion complex between hydroxypropyl-beta-cydodextrin vitamin E [58]. The prepared vitamin E-contained web provided enhanced photostability for the sensitive vitamin E by the inclusion complexation even after exposure to UV light. 

Based on previous reports, we have devoted our efforts to solving the increasingly serious azo dye contamination by novel selective composite fiber absorbents containing beta-cyclodextrin-based polymer (PCD) and ε-polycaprolactone (PCL). The electrospinning approach was an eco-friendly and simple preparation method. One of the indispensable advantages of the electrospinning membrane was the ultra-high specific surface area, which was extremely beneficial to adsorption. More importantly, the PCD was selected for its infinite long-chain and cavity structures. The obtained membrane contributed to the formation of more host–guest interaction due to a large number of free cyclodextrin cavities in the fiber surface. Thus, the excellent selective adsorption capability was foreseeable according to a previous report [61]. A few cyclodextrin cavities could be occupied by long-chain polymer molecules during the electrospinning progress, which is unfavorable for host–guest interactions and even results in a decrease in the undesirable adsorption effect. However, our PCL/(n%)PCD composite fibers have innumerable cavities, which could guarantee the selective adsorption capacity. Thus, it is obvious that the obtained PCL/(n%)PCD composite fibers can exhibit remarkable adsorption capacity towards azo dyes with the host–guest interaction. Moreover, the introduction of the β-cyclodextrin polymer could efficiently improve the mechanical strength and stability of the membrane. This indicated that the obtained composites have great potential to provide assistance with the problem of azo dye pollution in wastewater treatment. 

## 2. Materials and Methods

### 2.1. Materials

Beta-cyclodextrin (98%, abbreviated as β-CD) and epichlorohydrin (C_3_H_5_ClO, 99.5%) were purchased from Aladdin Chemicals (Shanghai, China). Methylbenzene (99%), chloroform (99%), and N,N-dimethylformamide (99%, abbreviated as DMF) were obtained from Beijing Chemicals (analytical reagent grade, Beijing, China). Acetone (C_3_H_6_O, 99.5%) was purchased from Alfa Aesar Chemicals (Shanghai, China). ε-Polycaprolactone (PCL average Mw~80000), methylene blue (MB) and 4-aminoazobenzene were purchased from Sinopharm Chemical Reagent Co., Ltd. (analytical reagent grade, Shanghai, China). Hydrochloric acid (HCl, 99%) and sodium hydroxide (NaOH, 99%) were obtained from Tianjin Kaitong Chemicals (Tianjin, China). Ultra-pure water was obtained using a Millipore Milli-Q water purification system with a resistivity of 18.2 MΩ·cm^−1^. All chemicals were used as received without further purification.

### 2.2. Preparation of β-Cyclodextrin Polymer (PCD)

First, the used β-cyclodextrin polymer (PCD) was synthesized as in previous similar studies [62,63]. In brief, 10 g β-cyclodextrin was dissolved in 15 mL aqueous 15 wt% NaOH solution in a clean beaker, and the system was stirred by mechanical agitation for at least 24 h at 35 °C in a water bath. Subsequently, the 2 mL toluene solution was added to a beaker that was continuously stirred at 35 °C for two hours. Then we added 14.8 mL epichlorohydrin solution and the whole system was stirred for 3 h. After that the mixture system was added to 200 mL acetone solution and stirred at 50 °C overnight. The precursor was filtered and dissolved in water, before using hydrochloric acid to neutralize it. After seven days of dialysis with ultra-pure water, freeze-drying treatment at −48 °C was performed. The product, solid white PCD, was obtained and stored for further use. 

### 2.3. Preparation of Electrospun Composite Fibers 

The total mass of the electrospinning precursor was 10 g. The mixture solvent was made of chloroform and N,N-dimethylformamide with a volume ratio of 3:2. The 1.2 g ε-polycaprolactone in pellet form and 8.8 g mixture solvent were magnetically stirred for 4 h to obtain a uniform solution, in accordance with previous reports [64,65]. Through electrospinning, neat PCL fibers were obtained. In addition, a different mass of poly β-cyclodextrin powder was added to a PCL/(CCl_4_/DMF) solution and formed a uniform spinning solution by magnetic stirring all night. During the following electrospinning, the flow rate was delivered at 1 mL·h^−1^, while the potential difference was set to 15–30 kV and the distance was 15–30 cm from the point of the needle to the collector. By regulating the spinning conditions, we finally obtained the optimal conditions based on the analysis of SEM images. On the condition of constant content of PCL molecules in total mass, different masses of poly β-cyclodextrin (PCD) component (10, 20, 30, 40, 50 wt%) were mixed with PCL to obtain composite fibers abbreviated as PCL/(n%)PCD (*n* = 10, 20, 30, 40, and 50). The specific components and quantities of electrospinning solution in the different groups are shown in Table 1. All of the samples were rested in a vacuum drying oven for two days in order to volatilize the remaining solvent.

### 2.4. Dye Removal Tests

The dyes methylene blue (MB) and 4-aminoazobenzene were used to estimate the adsorption properties of PCL/(n%)PCD (*n* = 10, 20, 30, 40, and 50) composite fibers with neat PCL fiber as the control group. UV–VIS absorption spectra were recorded for the process at wavelengths of 632 nm (MB) and 375 nm (4-aminoazobenzene) by a UV–VIS spectrometer. The freshly prepared definite samples (5 mg) were added to 50 mL dye solutions that contained MB (10 mg/L) and 4-aminoazobenzene (20 mg/L), respectively. The absorbance was measured, and corresponding concentrations and kinetic data were calculated by calibration curves. At the end of the adsorption process, all samples were washed with ethanol and DI water for several times and dried in a drying oven before further use. In addition, the recycling capacity of PCL/(n%)PCD composites was investigated. The prepared samples were repeatedly used to remove the same fresh MB solution for eight consecutive cycles.

### 2.5. Characterization

The microstructures of all the obtained composite materials were characterized by a field-emission scanning electron microscopy (FE-SEM, Hitachi S-4800-II, Tokyo, Japan) with 5–15 kV accelerating voltage. Energy-dispersive X-ray spectrometry (EDXS) was utilized to distinguish the elements in membranes at an accelerating voltage of 200 kV by taking advantage of an Oxford Link-ISIS X-ray EDXS microanalysis system. In addition, themogravimetry-differential scanning calorimetry (TG-DSC) was carried out to estimate the thermal stability of samples in air with a NETZSCH STA 409 PC Luxxsi multaneous thermal analyzer (Netzsch Instruments Manufacturing Co., Ltd., Seligenstadt, Germany). FTIR spectra were measured to analyze the molecular absorption spectroscopy by a Fourier infrared spectroscopy (Thermo Nicolet Corporation, Madison, WI, USA) using the KBr tablet method. X-ray diffraction (XRD) analysis was performed on an X-ray diffractometer equipped with a Cu Kα X-ray radiation source and a Bragg diffraction setup (SMART LAB, Rigaku, Japan). Circular dichroism spectra were measured by a JASCO J-810 CD spectrometer (Jasco Inc., Easton, MD, USA). UV–VIS absorption was used to monitor the adsorption progress by a UV–VIS spectrometer (752-type, Sunny Hengping Scientific Instrument Co., Ltd., Shanghai, China) at room temperature.

## 3. Results and Discussion

### 3.1. Structural Characterization of the Composite Polymer Fibers

**Figure 1:**
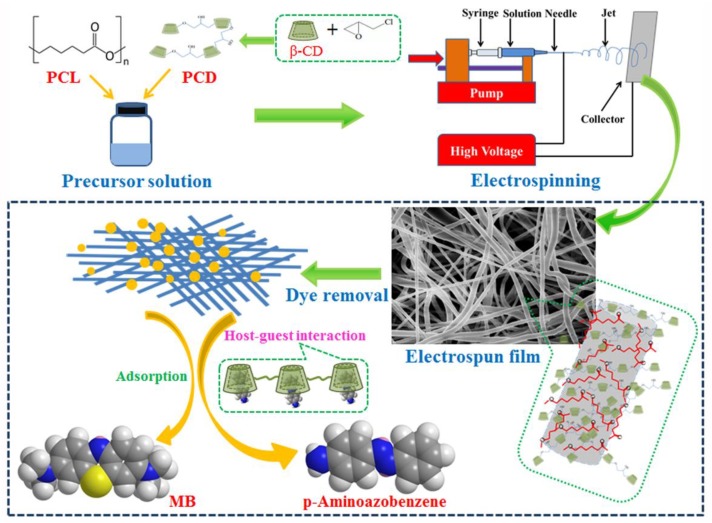
Schematic illustration of preparation and application in dye removal of PCL/(n%)PCD composite fibers by electrospinning.

Firstly, we prepared a uniform PCL spinning solution by taking advantage of the CCl_4_ and DMF mixed solvent. By attempting different parameters including voltage, type of stainless steel needle, distance between needle, aluminum foil, and injection rate, we finally confirmed the optimal conditions that need to be abided by in the following electrospinning. The illustration of the preparation and application in organic dyes of PCL/(n%)PCD composites is shown in Figure 1. Thus, we can get the PCL/(n%)PCD composite fibers by electrospinning under the optimal parameter conditions. In order to volatilize the excess solvent, all the samples have been put in a drying oven for two days. The adsorption capacities of a series of composite fibers were characterized by taking advantage of the methylene blue (MB) and 4-aminoazobenzene solution. 

**Figure 2:**
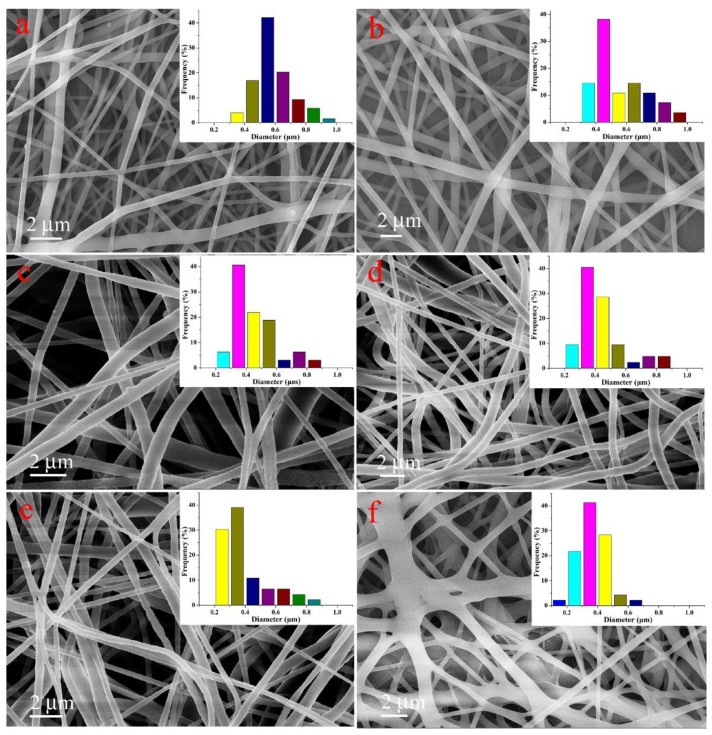
SEM images and diameter distribution histograms of neat PCL fibers (**a**), PCL/(10%)PCD (**b**), PCL/(20%)PCD (**c**), PCL/(30%)PCD (**d**), PCL/(40%)PCD (**e**), and PCL/(50%)PCD (**f**).

The optimal conditions of electrospinning were obtained under different mixed ratios of PCD, after attempts and characterization of different parameters. Based on this, the representative micromorphology images by SEM of neat PCL fibers and PCL/(n%)PCD composite fibers are depicted in Figure 2. The neat PCL fibers showed a homogeneous solid fiber structure, and multiple layers of fibers were stacked together in the form of an electrospun membrane. The physical properties of the spinning precursor solution changed through different ratios of PCD power. Therefore, the parameters of the electrospinning have also changed. After multiple trials and adjustment, the ideal conditions for fiber formation were obtained. The micromorphology pictures of fibers with different ratios of PCD, that is, PCL/(10%)PCD, PCL/(20%)PCD, PCL/(30%)PCD, PCL/(40%)PCD, and PCL/(50%)PCD, can be seen in Figure 2. The diameter of neat PCL fibers appeared at a centered position of 500–600 nm with the length in microns. In addition, with the addition of a PCD component in fibers, the fiber diameters decreased and reached a centered range of 200–400 nm for the obtained PCL/(40%)PCD composite fiber. A possible reason for diameter decrement was that the strong network between the neighboring chains could be temporarily destroyed during the electrospinning process, which enhanced the stretching of the jet [66]. As for the PCL/(50%)PCD composite fiber, more cross-linking fibers could be clearly observed, mainly due to the increasing viscosity of the precursor solution. Moreover, when the content of PCD in precursor solution exceeded 50%, the electrospun needle would clog and could not obtain continuous electrospun fibers.

**Figure 3:**
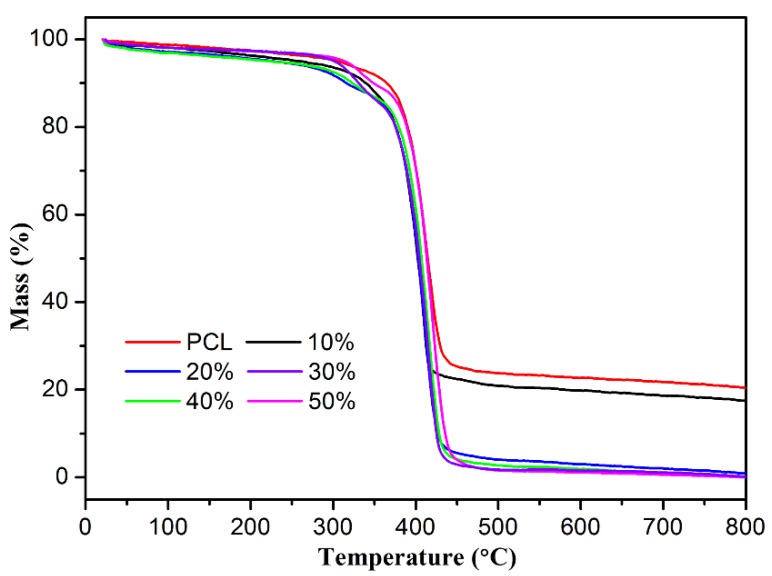
TG curves of neat PCL fibers and composite fiber composites.

It was well known that the thermal stability of composite materials is an important factor in their characterization and wider application. The thermal stabilities of the obtained neat PCL fiber and a series of PCL/(n%)PCD fiber composites were investigated by the thermogravimetry (TG) curves as shown in Figure 3. In the N_2_ condition, all the samples were heated from room temperature to 800 °C through a temperature-programmed route. Before the temperature reached 300 °C, the thermogravimetric curves of PCL fiber remained stable and there was no significant weightlessness. The one-stage degradation was related to the decomposition of the carbon skeleton from 372 °C to 457 °C. The final weight loss was approximately 79.8 wt% at 800 °C. In the cases of present PCL/(n%)PCD composites, weight loss below 150 °C could be considered as removal of trace moisture vapor adsorbed by PCD fibers and/or a small amount of crystal water entrapped by PCD cavities. After that, the thermal degradation from 307 °C to about 370 °C corresponds to PCD molecules. This is followed by the degradation of neat PCL fibers from about 370 °C to 450 °C. With the increment of the PCD component, the initial degradation temperature and the final mass of residue of PCL/(10%)PCD obviously dropped to 17.4 wt%, while the other PCL/(n%)PCD composites was about 3 wt%. It was obvious that the weight loss originated from decomposition of PCL and PCD components. 

**Figure 4:**
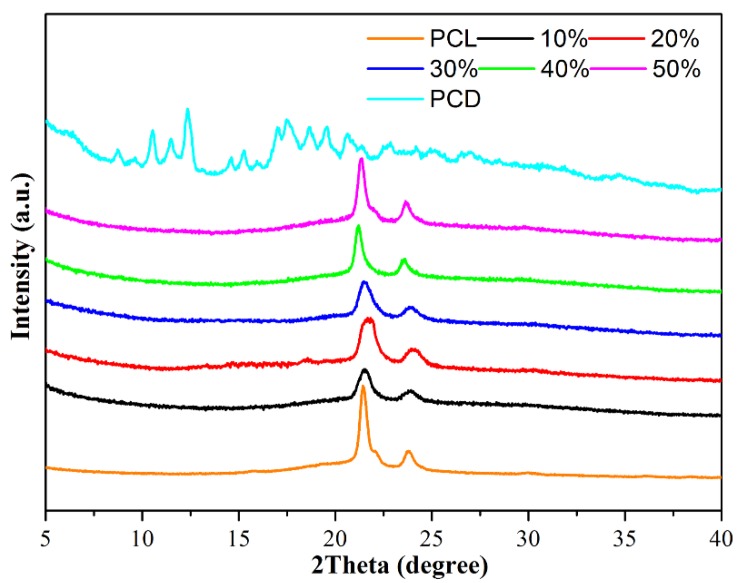
XRD patterns of the prepared neat PCL fibers and composite fiber composites.

XRD data are frequently used to characterize and confirm the presence of both components. As seen in Figure 4, the two diffraction peaks of neat PCL fibers appeared at 2θ values of 22° and 24° can be indexed to (110) and (200) reflections, which confirms its orthorhombic crystal structure. In addition, PCD powder elicits many small and cluttered characteristic peaks, which can be attributed to the cage structure of native beta-CD. It was apparent that all samples of obtained PCL/(n%)PCD composite fibers show the same characteristic peak and only have two components without any other impurities. However, slightly broadened characteristic peaks of PCL molecules in PCL/(n%)PCD fibers can be clearly observed. We also noted that the peak shifted slightly to the right. Such results can imply that there are some interactions between PCL and PCD molecules. The content of PCD incorporated into PCL fibers was little in our work. Therefore, it is not sufficient to cause the obvious XRD spectral characteristic peak change of PCL/(n%)PCD composite fibers. XRD results complement the TG findings and indicate the presence of a physical mixture in the obtained composites as well.

**Figure 5:**
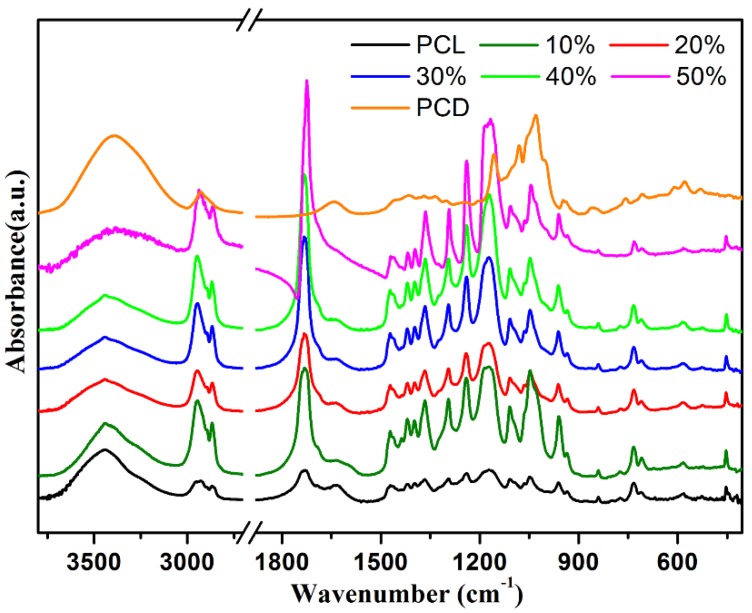
FT-IR spectra of neat PCL fibers and PCL/(n%)PCD (*n* = 10, 20, 30, 40, 50) composites.

FT-IR analyses were performed for conclusive evidence, and the results are shown in Figure 5. The conformational changes of the PCL fibers and PCL/(n%)PCD fibers were characterized by Fourier transform infrared spectroscopy. It can be seen that the characteristic peaks of pure PCL spectra are mainly typical ester bonds and hydrocarbon bonds. The additional peaks at 1728 cm^−1^, 1243 cm^−1^, and 1046 cm^−1^ represent the vibration peaks of C=O, C–O–C, and C–C groups. In addition, the strong wide peak at 3355 cm^−1^ can be ascribed to the association of hydrogen bonds formed by the –OH group. In addition, the absorption peak at 1033 cm^−1^ represents C–O–C and C–O stretching vibration of the beta-CD cross-linked polymer cavity. In addition, composite fibers of PCL with increasing content of PCD addition (0, 10, 20, 30, 40, and 50 wt%) can show similar changes of type and position of characteristic peak. Based on the above, the designed PCL/(n%)PCD composite fiber samples were successfully synthesized. In addition, the microstructures of the obtained PCL fiber and PCL/(n%)PCD composite fibers were investigated using N_2_ adsorption–desorption isotherms. The obtained properties of the samples were generalized in Table 2. It could be clearly observed that the as-obtained PCL fibers showed a specific surface area of 7.50 m^2^·g^−1^. In addition, with the increment of the PCD component in the composite fibers, the values of specific surface area obviously increased and reached 11.52 m^2^·g^−1^ for PCL/(50%)PCD composite fibers, demonstrating the formation of more anchoring sites facilitating the next adsorption of dye molecules. Meanwhile, the pore size and pore volume of all samples were calculated via BJH methods. The obtained PCL/(50%)PCD composite fibers also exhibited enhanced pore size and pore volume, meaning that larger pore diameters and pore volumes in composite fibers could demonstrate lots of micro/nanoscale channels, thereby making them effective for the next adsorption experiment.

**Figure 6:**
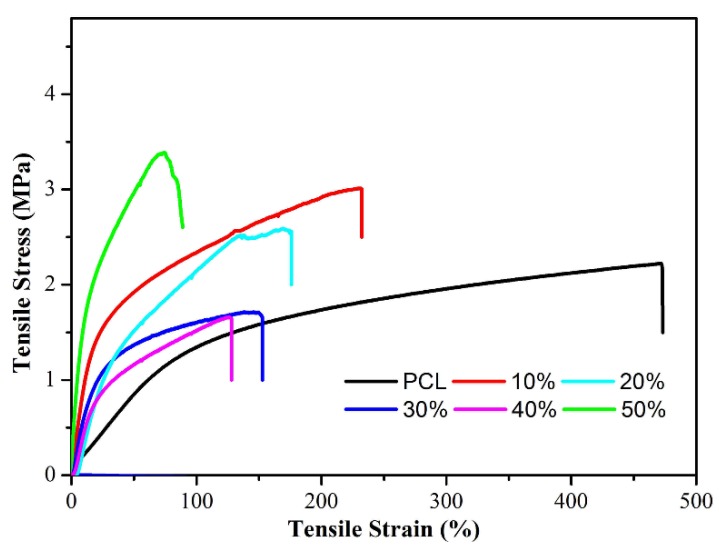
Stress–strain plots of neat PCL fibers and PCL/(n%)PCD (*n* = 10, 20, 30, 40, 50) composites.

The tensile properties and stress–strain plots of several PCL/(n%)PCD composites were conducted at room temperature with neat PCL fibers as a control for comparison, as shown in Figure 6. It is evident that the neat PCL fiber has a high elongation at break (above 470%). Correspondingly, the elongation at break of our functionalized PCL/(n%)PCD samples has decreased markedly with the increase in PCD content. The elongation of PCL/(10%)PCD fibers at break was 230%, while the value of PCL/(50%)PCD fibers was only 74%. The elongation at break of PCL/(20%)PCD, PCL/(30%)PCD and PCL/(40%)PCD was 171%, 150%, and 127%, respectively. Compare with neat PCL fibers, the elongation at break eventually declined sharply by six times. In addition, the ultimate tensile strength of neat PCL was 2.25 MPa. In hybridization cases of PCL/(n%)PCD samples, the fracture stress presented a trend of first increasing, then decreasing, and next increasing along with the change of content of PCD. It could be seen that the fracture stress generally increased and the final PCL/(50%)PCD composites were destroyed when the fracture stress reached 3.41 MPa. Clearly, the introduction of PCD significantly weakened the elongation at break but prominently increased the ultimate tensile strengths of present composite fibers. This change phenomena seemed similar to previous report about electrospun composite poly(ethylene glycol)/poly(caprolactone) nanofibrous membrane [67]. One explanation could be that the content of PCD increased with the decrease of solvents, resulting in the presence of hard segments or clusters. It could be clearly seen that the obtained PCL/(n%)PCD composite materials showed good tensile strength, with PCD component playing a key role in improving it [68,69].

### 3.2. Dye Removal Performance of the Composite Fibers

**Figure 7:**
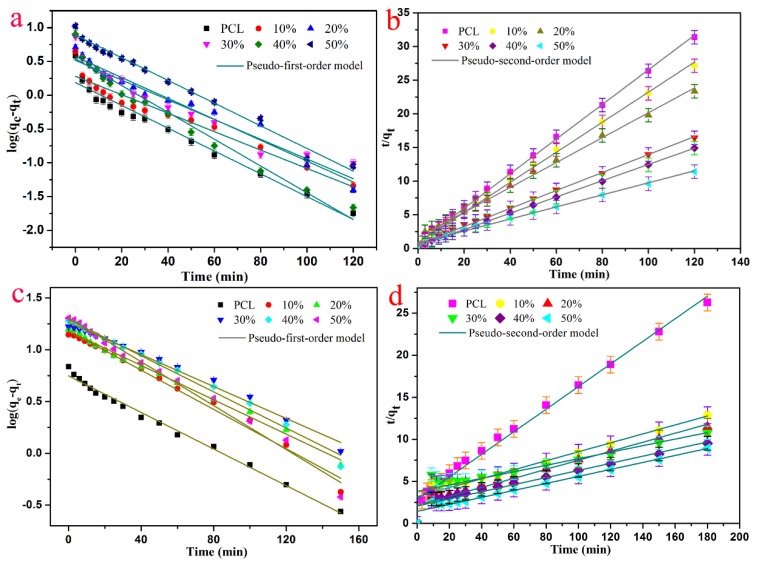
Kinetic adsorption of MB (**a**,**b**) and 4-aminoazobenzene (**c**,**d**).

In order to characterize the selective dye removal performances of the present obtained composite fiber absorbents, the obtained PCL/(n%)PCD composite fibers were investigated with respect to their uptake of removing MB and 4-aminoazobenzene (AA) as typical models [70,71]. The mechanism of removing organic dye molecules was adsorption and host–guest interaction for MB and 4-aminoazobenzene, respectively. In addition, neat PCL was used as a control group and the whole adsorption progress was monitored by taking advantage of the UV–VIS spectra. The adsorption properties of PCL/(n%)PCD composites toward MB and 4-aminoazobenzene are shown in Figure 7. Clearly, the PCL/(n%)PCD samples showed better dye uptake than neat PCL toward the two organic dyes. However, the adsorption uptake of PCL/(n%)PCD composite fibers became better with the increment of the content of PCD compared with PCL. The adsorption kinetics data distinctly demonstrated the above view. The pseudo-first-order model and pseudo-second-order model adsorption equations were used to further evaluate adsorption kinetics by fitting the experimental data. All the fitted results are summarized in Table 3.

The pseudo-first-order model can be demonstrated by Equation (1) [53]:

(1) $$\log\;\left(q_{e}-q_{t}\right)=\;\log\;q_{e}-\frac{k_{1}}{2.303}\;t$$

where *t* is the adsorption time, *q_e_* is the adsorption capacity at equilibrium, *k*_1_ is the pseudo-first-order model rate constant, and *q_t_* is the adsorption capacity at time *t*.

The pseudo-second-order model can be demonstrated by Equation (2) [72]:

(2) $$\frac{t}{q_{t}}=\frac{1}{k_{2}q_{e}{}^{2}}+\frac{t}{q_{e}}$$

where *q_e_* is the adsorption uptake at equilibrium, *k*_2_ is the pseudo-second-model rate constant, and *q_t_* is the adsorption uptake at time *t*.

In the case of the MB solution, the pseudo-second-order model had a higher correlation coefficient (*R*^2^ > 0.99) than the pseudo-first-order model (*R*^2^ > 0.93). The obtained values of adsorption uptake were almost equal to those fitted from the pseudo-second-order model. In addition, neat PCL fibers showed low adsorption uptake, while the PCL/(n%)PCD samples all greatly improved, as shown in Figure 7 and Table 2. The dye removal efficiency of neat PCL fibers only reached 3.8246 mg/g. With the increment of PCD content, the adsorption uptake of composite fibers enhanced significantly, from 4.4238 mg/g to 10.5238 mg/g. The composite fibers showed a good adsorption performance for MB mainly due to the following two reasons. Firstly, PCL, as the most basic component in electrospun fibers, has almost no efficient groups for adsorption. Secondly, the unique cavity structure of PCD has great potential to identify and select organic molecules. Thus, MB molecules could not enter into the cavity of PCD. Only a small number of hydroxyl groups could form hydrogen bonds to facilitate the adsorption of the MB dye solution. However, we could still come to the conclusion that the introduction of PCD was conducive to adsorption.

By contrast, PCL/(n%)PCD samples exhibited outstanding adsorption uptake to 4-aminoazobenzene. It was clearly observed that the pseudo-second-order (*R*^2^ > 0.99) was more accurate than the pseudo-first-order model. Thus, the adsorption progress was more consistent with the pseudo-second-order dynamic model. In Table 3, the dye removal efficiency of PCL/(10%)PCD could reach 18.7512 mg/g, which was twice as high as the PCL fibers’ adsorption uptake. While more and more PCD molecules were added and the proportion in spinning membranes was increasing, the dye removal efficiency of the obtained composite fibers increased continuously and rapidly. The PCL/(50%)PCD composites finally reached 24.0674 mg/g. Apparently, the adsorption uptake of PCL/(n%)PCD toward 4-aminoazobenzene had appreciable performance due to the addition of host–guest inclusion complexation. The 4-aminoazobenzene molecules could be included in the cavity structure of PCD through host–guest interaction and/or being bound by the fiber surface through electrostatic interaction and hydrogen bonds. The formed self-assembled structures were stable and highly efficient.

**Figure 8:**
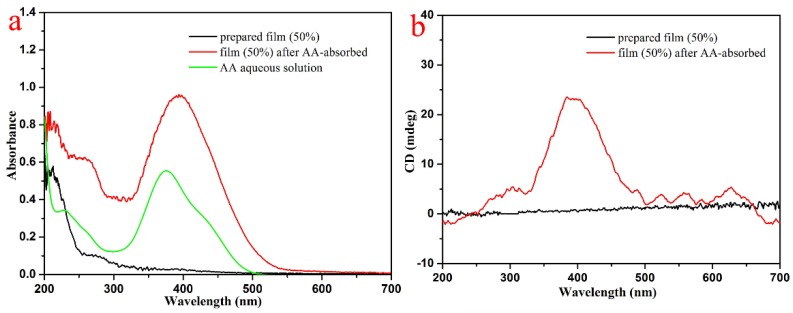
UV–VIS (**a**) and circular dichroism spectra (**b**) of the PCL/(50%)PCD composite fibers.

**Figure 9:**
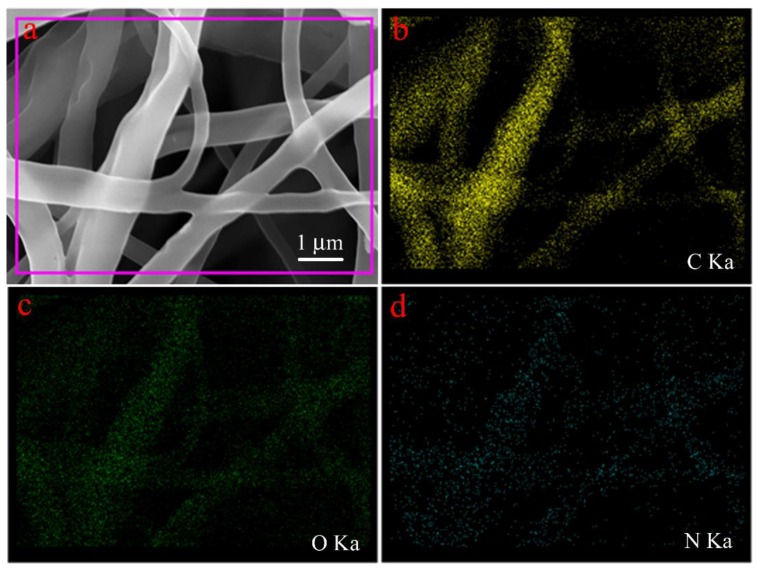
SEM image (**a**) with C/O/N elemental mapping (**b**–**d**) of AA-absorbed PCL/(50%)PCD composite fibers.

In order to further prove that the driving force of removal mechanism relative to 4-aminoazobenzene consisted of host–guest interaction, we collected the data of UV–VIS and circular dichroism spectra to characterize the PCL/(50%)PCD membrane before and after the adsorption process, as shown in Figure 8. Obviously, as-prepared PCL/(50%)PCD membrane have no significant characteristic peak in Figure 8a. After adsorption of 4-aminoazobenzene, the maximum absorption peak of the composites appears at 400 nm, which was attributed to π–π* electron transition of the 4-aminoazobenzene group [73,74,75,76,77]. However, the additional characteristic peak of 4-aminoazobenzene was at 370 nm. Therefore, the peak position of PCL/(50%)PCD membrane had a red shift after adsorption, which could result from an interaction between the hydroxyl of PCD and the chromogenic group of 4-aminoazobenzene. The circular dichroism spectra of PCL/(50%)PCD membrane have shown similar results. The intensity of the signal was notable at 400 nm, with one positive Cotton effect in Figure 8b. In addition, the images of SEM with C/O/N elemental mapping of PCL/(50%)PCD composite fibers after adsorption of 4-aminoazobenzene have also been measured and are shown in Figure 9. Obviously, a large quantity of N element was well distributed onto the obtained fibers (Figure 9d), which further confirmed the presence and the good distribution of 4-aminoazobenzene in the obtained composite fiber. It could be speculated that hydrophilic 4-aminoazobenzene molecules was successfully anchored on the surface of PCL/(50%)PCD fibers by intermolecular host–guest interaction and/or electrostatic interaction/hydrogen bonding, which could be expected to exert adsorption activity and good stability in the next recovery and reuse process. Thus, combined with UV–VIS spectra, the presence of a 4-aminoazobenzene group in the obtained composite materials was further confirmed. So, it could be considered that the host–guest reaction occurred and the 4-aminoazobenzene moiety was located inside the cavity of PCD molecules via host–guest interaction and/or anchored the surface of fiber via electrostatic interaction/hydrogen bonding. In addition, it should be noted that the signal intensity of the circular dichroism spectra was lower, which could be mainly due to two reasons. Firstly, partial PCD did not have enough contact with 4-aminoazobenzene to form an inclusion complex because some cavities of PCD were inside the fibers. Secondly, partial 4-aminoazobenzene molecules only stayed on the surface of the membrane due to intermolecular hydrogen bonding.

**Figure 10:**
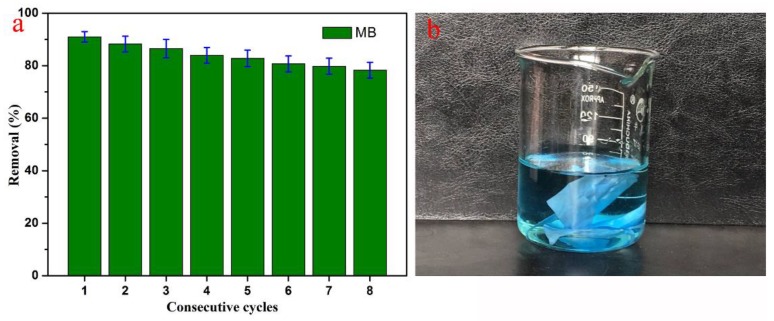
Relative adsorption reutilization studies towards removal of MB for different consecutive cycles at room temperature of PCL/(50%)PCD composites (**a**) and the photograph of PCL/(50%)PCD composite after eight reutilization cycles (**b**).

Adsorption reutilization research into PCL/(50%)PCD’s removal of MB was conducted and the results are shown in Figure 10, with graphic illustration of used nanocomposite after eight reutilization cycles. The obtained PCL/(n%)PCD composite fibers could be recovered and reused by a simple washing and drying process. The membrane became blue after the adsorption of MB molecules, followed by desorption in ethanol. Rapid and simple desorption and regeneration created favorable conditions for reutilization. In the case of PCL/(50%)PCD, the removal of MB dye could still reach 78% after repeated adsorption over eight cycles. The loss of adsorption may result from a slightly deformed fiber structure and a few cavities of PCD molecules being occupied. All in all, the composites consisting of PCL and PCD molecules have good stability in the field of adsorption of organic dyes. It should be noted that the obtained PCL/(n%)PCD composites can hardly exhibit outstanding adsorption capacity in a three-dimensional matrix such as a hydrogel structure. However, the present PCL/(n%)PCD composites have demonstrated a capacity for selectivity adsorption and excellent stability towards present two kinds of dyes. In addition, the electrospinning nanocomposites formed by poly β-cyclodextrin have relatively more cavities than β-cyclodextrin monomer molecules. Thus, generous cavities were highly beneficial to the field of selectivity adsorption. Moreover, the introduction of PCD molecules made the composites have better mechanical strength, which also contributed to the improvement of stability. All in all, the PCL/(n%)PCD electrospinning membranes not only have remarkable selectivity adsorption, but also admirable stability, which is important for the prospect of industrialization in the field of wastewater treatment and self-assembled nanomaterials [78,79,80].

## 4. Conclusions

In summary, we have successfully prepared electrospun PCL/(n%)PCD (*n* = 10, 20, 30, 40, 50) composite fiber materials via a simple and low-cost method. It could be seen that the prepared PCL/(n%)PCD composites showed uniform fiber nanostructures and had been well characterized. According to the strain–stress plots, PCD molecules were advantageous to improve the mechanical strength of the obtained fiber films. In addition, the introduction of PCD led to excellent uptake of selectivity adsorption in the obtained electrospun composite films with a high specific surface area. In addition, the improvement of mechanical strength also enhances the stability of electrospinning membranes. This research work has proposed a new design of electrospun composites with a cyclodextrin component and suggested new possibilities in selective adsorption for dye removal.

## Figures and Tables

**Table 1:** The specific components and quantities used in electrospinning precursor solutions.

PCD Concentration (wt%)	0	10	20	30	40	50
PCL (g)	1.20	1.20	1.20	1.20	1.20	1.20
CCl_4_ (mL)	4.17	4.11	4.02	3.92	3.78	3.60
DMF (mL)	2.78	2.74	2.68	2.61	2.52	2.40

**Table 2:** Physical data of as-prepared composite fibers.

Samples	Specific Surface Area (m^2^·g^−1^)	Average Pore Diameter (nm)	Pore Volume (cm^3^·g^−1^)
PCL fiber	7.50	20.1	0.008231
PCL/(10%)PCD	7.82	20.5	0.008464
PCL/(20%)PCD	7.96	21.0	0.008576
PCL/(30%)PCD	8.45	22.5	0.009125
PCL/(40%)PCD	10.85	23.6	0.009277
PCL/(50%)PCD	11.52	23.6	0.009446

**Table 3:** The fitting results achieved for kinetic adsorption data using the pseudo-first-order model and pseudo-second-order model equation.

**MB**	**Pseudo-First-Order Model**			**Pseudo-Second-Order Model**		
	***q**_**e**_* **(mg/g)**	***R*^2^**	**K_1_** **(min^−1^)**	***q_e_*** **(mg/g)**	***R*^2^**	**K_2_** **(g/mg min)**
PCL	3.8246	0.9337	0.0393	3.8879	0.9989	0.0811
PCL/(10%)PCD	4.4238	0.9129	0.0317	4.4767	0.9979	0.0568
PCL/(20%)PCD	5.1414	0.9498	0.0337	5.4077	0.9936	0.0206
PCL/(30%)PCD	7.3002	0.9404	0.0376	7.4862	0.9980	0.0303
PCL/(40%)PCD	8.0465	0.9398	0.0550	8.2740	0.9991	0.0373
PCL/(50%)PCD	10.5238	0.9837	0.0399	11.1632	0.9931	0.0102
4-aminoazobenzene	Pseudo-first-order model			Pseudo-second-order model		
	*q_e_* (mg/g)	*R* ^2^	K_1_ (min^−1^)	*q_e_* (mg/g)	*R* ^2^	K_2_ (g/mg min)
PCL	6.8517	0.9916	0.0203	7.4427	0.9868	0.0064
PCL/(10%)PCD	13.9828	0.9872	0.0218	18.7512	0.9981	0.0090
PCL/(20%)PCD	15.6643	0.9941	0.0191	18.6324	0.9931	0.0014
PCL/(30%)PCD	16.7213	0.9986	0.0178	25.7070	0.9986	0.0004
PCL/(40%)PCD	18.9147	0.9895	0.0201	23.6183	0.9978	0.0009
PCL/(50%)PCD	20.1740	0.9870	0.0244	24.0674	0.9943	0.0012
